# Climate Variability and Oceanographic Settings Associated with Interannual Variability in the Initiation of *Dinophysis* *acuminata* Blooms

**DOI:** 10.3390/md11082964

**Published:** 2013-08-16

**Authors:** Patricio A. Díaz, Beatriz Reguera, Manuel Ruiz-Villarreal, Yolanda Pazos, Lourdes Velo-Suárez, Henrick Berger, Marc Sourisseau

**Affiliations:** 1 Spanish Institute of Oceanography (IEO), Oceanographic Centre of Vigo, Subida a Radiofaro 50, Vigo 36390, Spain; E-Mail: beatriz.reguera@vi.ieo.es; 2 Spanish Institute of Oceanography (IEO), Oceanographic Centre of A Coruña, Muelle de las Ánimas s/n, A Coruña 15001, Spain; E-Mail: manuel.ruiz@co.ieo.es; 3 Technological Centre for the Control of the Marine Environment of Galicia (INTECMAR), Peirao de Vilaxoán s/n, Vilagarcía de Arousa 36611, Pontevedra, Spain; E-Mail: ypazos@intecmar.org; 4 French Research Institute for the Exploitation of the Sea (IFREMER), Brest Centre, Pointe du Diable BP 70, Plouzané 29280, France; E-Mails: Lourdes.Velo.Suarez@ifremer.fr (L.V.-S.); Henrick.Berger@ifremer.fr (H.B.); Marc.Sourisseau@ifremer.fr (M.S.)

**Keywords:** *Dinophysis acuminata*, climate variability, upwelling patterns, river plumes, exceptional algal blooms, predictive models

## Abstract

In 2012, there were exceptional blooms of *D. acuminata* in early spring in what appeared to be a mesoscale event affecting Western Iberia and the Bay of Biscay. The objective of this work was to identify common climatic patterns to explain the observed anomalies in two important aquaculture sites, the Galician Rías Baixas (NW Spain) and Arcachon Bay (SW France). Here, we examine climate variability through physical-biological couplings, Sea Surface Temperature (SST) anomalies and time of initiation of the upwelling season and its intensity over several decades. In 2012, the mesoscale features common to the two sites were positive anomalies in SST and unusual wind patterns. These led to an atypical predominance of upwelling in winter in the Galician Rías, and increased haline stratification associated with a southward advection of the Gironde plume in Arcachon Bay. Both scenarios promoted an early phytoplankton growth season and increased stability that enhanced *D. acuminata* growth. Therefore, a common climate anomaly caused exceptional blooms of *D. acuminata* in two distant regions through different triggering mechanisms. These results increase our capability to predict intense diarrhetic shellfish poisoning outbreaks in the early spring from observations in the preceding winter.

## 1. Introduction

Endemic occurrence of *Dinophysis* species producers of lipophilic shellfish toxins (okadaic acid, dinophysistoxins, pectenotoxins) are the main cause of shellfish harvesting closures in Western Europe, in particular on the Atlantic coasts of Iberia [1,2], France [3,4], SW Ireland [5], Skagerrak-Kattegat [6], and Norway [7]. The areas under study are Arcachon Bay (SE Bay of Biscay), an important area for production of commercial oysters (*Crassostrea angulata/gigas*) and their seeds, and Ría de Pontevedra, one of the four Galician Rías Baixas (NW Spain), and a site of intensive raft mussel (*Mytilus galloprovincialis*) aquaculture.

Arcachon Bay is a shallow mesotidal lagoon with a maximal depth of 10 m and high exchange rates with the adjacent shelf waters [8]. Scattered cells of *D. acuminata* can be detected all year round in Arcachon Bay, but earlier studies have shown that dense populations of *Dinophysis* build up offshore and are advected into the bay before Diarrhetic Shellfish Poisoning (DSP) outbreaks occur [9]. Spring phytoplankton blooms in continental shelf waters close to Arcachon Bay usually start in March but earlier winter blooms have been observed under favourable conditions [10,11]. Bloom timing is associated with stratification which is usually thermal due to a northward advection of the Gironde River plume during the winter [12] and the absence of any other significant river. Typically, low thermal stratification appears by the end of April and local upwelling processes start in April and May. *Dinophysis* densities along the coast are usually low (<10^3^ cells L^−1^) and, as noted by Delmas *et al.* [13], are mainly associated with the persistence of stratification for a long period (>one month) and nutrient depletion. The usual timing for the first *Dinophysis* maximum in Arcachon Bay is late March. *Dinophysis* distribution from 2005 to 2008 along the shelf of Les Landes [9], a representative area for the south-western Bay of Biscay, showed large vertical heterogeneities. Observations of a maximum at depth suggest inoculum populations are located in the bottom layers. Because of the bathymetry of this open area (in contrast to the Galician Rías), local upwelling processes are only able to effectively introduce plankton populations in the coastal areas if stratification in the open waters and a double-layered circulation pattern are established.

The hydrodynamics of Ría de Pontevedra, located at the northern limit of the Canary Current Upwelling System, is mainly influenced by seasonal upwelling (spring to early autumn)—when prevailing northerly winds promote coastal upwelling of cold, saltier and nutrient-rich Eastern North Atlantic Central Water (ENACW)—and by coastal downwelling and freshwater inputs from October to March [14,15]. In the upwelling season, wind reversals lead to rapid changes in the hydrodynamics of the Rías and in plankton distributions [16,17]. In the Galician Rías Baixas, *D. acuminata* populations may occur throughout the upwelling season and the first maxima (>10^3^ cells L^−1^) appear in June [18]. In a conceptual model proposed earlier, the onset of the upwelling season and availability of *Mesodinium* prey are essential conditions for the initiation of *D. acuminata* blooms in the Galician Rías Baixas [18]. Thanks to monitoring inputs and previous research projects, considerable knowledge has been gained concerning seasonality and short-term response of *Dinophysis* spp. to environmental conditions. However, very little is known about the causes of their inter-annual variability. 

Single-species predictive models require a sound knowledge of the biology of the target organism. For example, in cyst-forming dinoflagellates, sexual cyst density in the top sediment layer is a very important parameter—the potential inoculum—used in forecast models [19]. Observations of planozygote division—with no need of sexual cyst maturation—and the rare occurrence of “putative cysts” (that turned out to be asexual cysts of *Fragilidium* recently fed on *Dinophysis* [1]) during exceptional blooms support the view that *Dinophysis* spp. do not rely on resting cysts germination as a seeding strategy [20]. Identification of the “pelagic seed beds” [21] and the factors controlling their introduction into the aquaculture sites in Galicia and SW France are therefore needed. 

**Figure 1:**
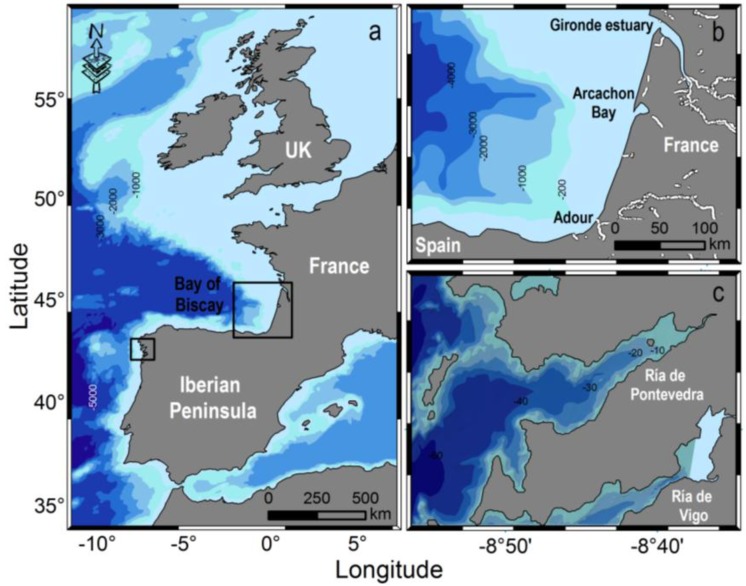
(**a**) Map of SW Europe showing the two study areas (rectangles); (**b**) Arcachon Bay and SE Bay of Biscay; (**c**) Ría de Pontevedra (NW Spain).

In 2012, there was an exceptionally early bloom of *D. acuminata* in terms of early initiation and high cell densities in the Galician Rías, and in terms of intensity in the southeast corner of the Bay of Biscay. These two distant areas have very different coastal morphology (Figure 1) and hydrodynamics but both have intensive aquaculture exploitations subject to recurrent DSP outbreaks caused by blooms of *Dinophysis*. This paper examines winter meteorological conditions and timing of initiation of *D. acuminata* blooms in a time series over several decades. The main objective was to identify common large scale meteorological conditions and environmental triggers explaining the exceptional bloom of *D. acuminata* in 2012.

## 2. Results

### 2.1. Meteorological and Hydrodynamic Conditions in the Two Study Areas in 2012

**Figure 2:**
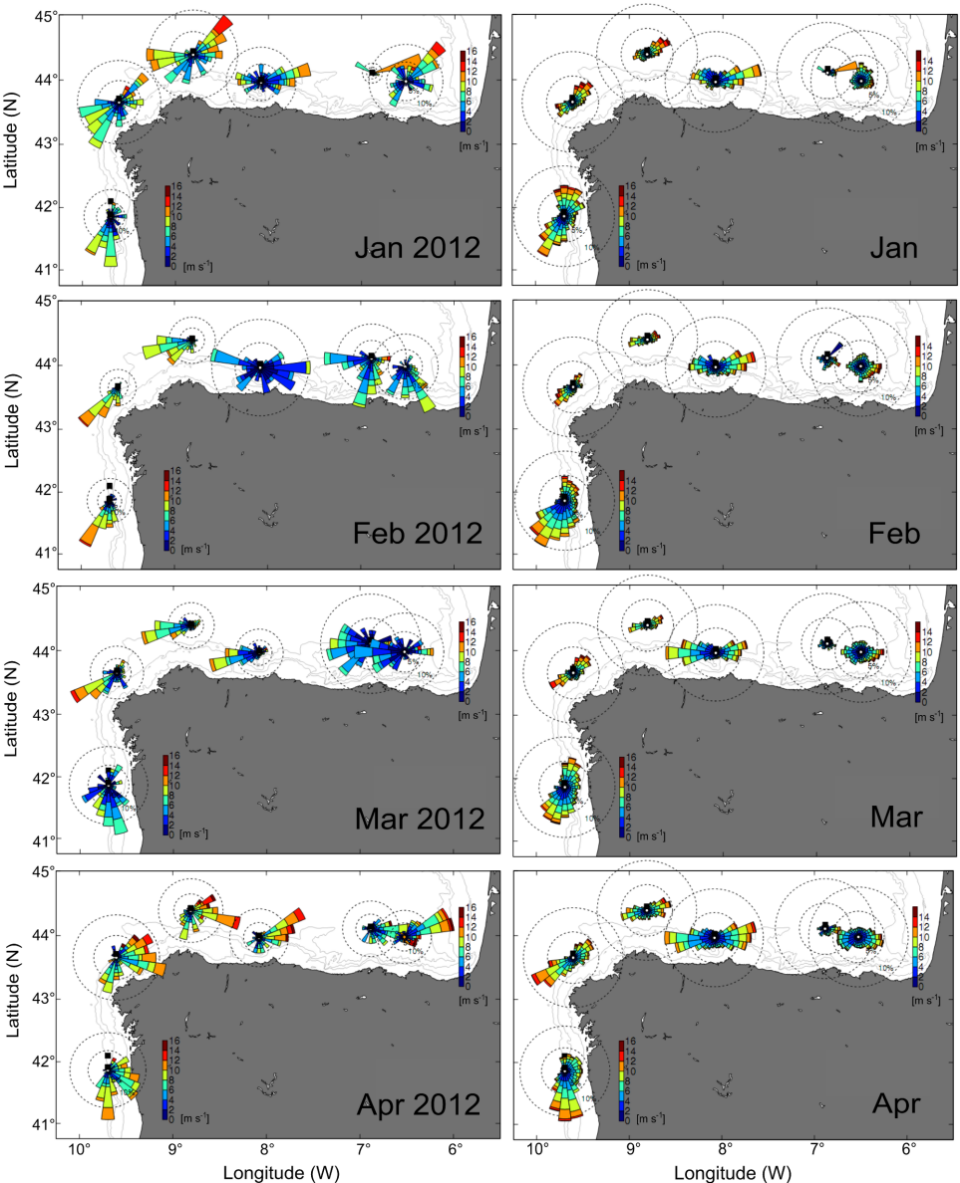
Comparison between wind conditions during winter-spring 2012 (left) and the climatology 1998–2012 (right) on the northwest and north Iberian Peninsula (winds directions are plotted following the oceanographic convention).

**Figure 3:**
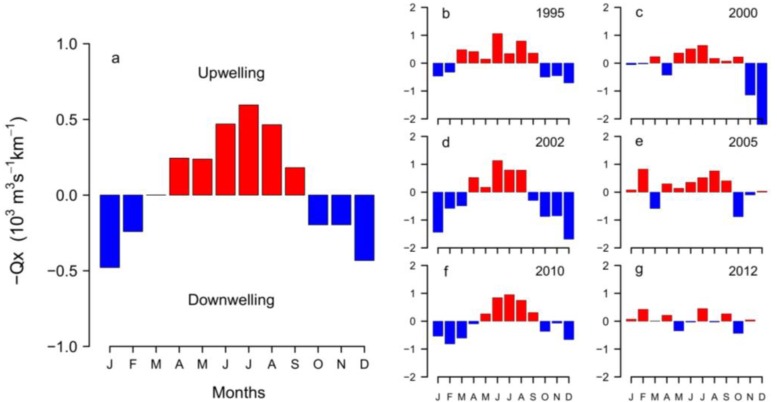
(**a**) Monthly average (1967–2011) Ekman transport (m^3^ s^−1^ km^−1^) estimated from the FNMOC-model; (**b**–**g**) Monthly Ekman transport of selected years with normal (1995, 2002 and 2010) and anomalous (2000, 2005 and 2012) upwelling patterns in relation to the historical monthly average in the Galician Rías Baixas.

**Figure 4:**
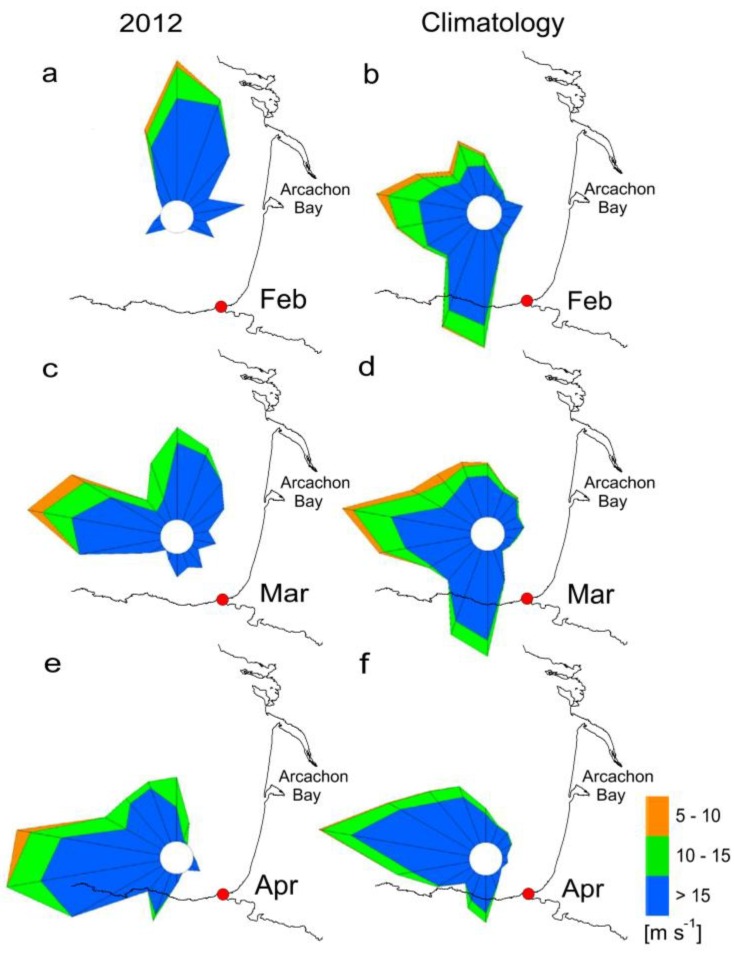
Comparison between wind conditions (**a**, **c**, **e**) during winter-spring 2012 and (**b**, **d**, **f**) the climatology in Arcachon Bay. Location of the Pointe de Socoa Meteorological Station is marked with a red dot (wind directions are plotted following the meteorological convention).

Unusual winds patterns in northwest Spain in early 2012 led to anomalous positive Ekman transport in winter in the Galician Rías. Likewise, in the Cantabrian Sea (southern Bay of Biscay) coast an unusual dominance of easterly winds was observed during this period (Figure 2). Usually, winters in the Rías are characterized by a predominance of southerly winds and negative Ekman transport followed by an upwelling-season from April to October, as shown by the 28-year means for the region (Figure 3a). Nevertheless, an atypical pattern of positive Ekman transport characterized by upwelling-dominance during the winter of 2012 was observed (Figure 3g). Similar anomalies, with a predominance of northerly winds in late winter, were also observed in south-western France, where southerly and westerly winds are dominant in this season (Figure 4). 

**Figure 5:**
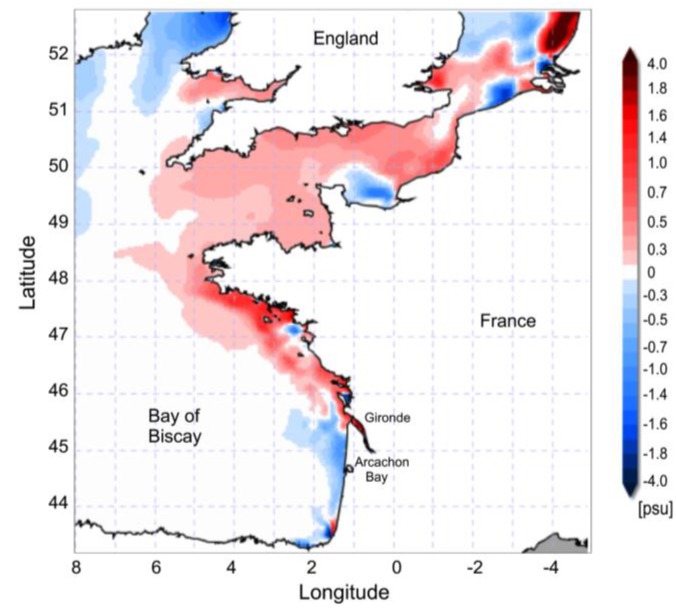
Sea surface salinity (SSS_2012_–SSS_Ref_) anomalies simulated in February 2012 by the French operational model (MARS-3D, Ifremer) over shelf waters of the eastern Bay of Biscay and the English Channel region. The reference field was estimated from 2010 to 2011.

Northerlies caused a deflection of the Gironde River plume to the southern part of Biscay, where strong negative salinity anomalies were inferred, according to simulations of the MARS-3D hydrodynamic model (Figure 5), in spite of lower freshwater inputs than in previous years.

**Figure 6:**
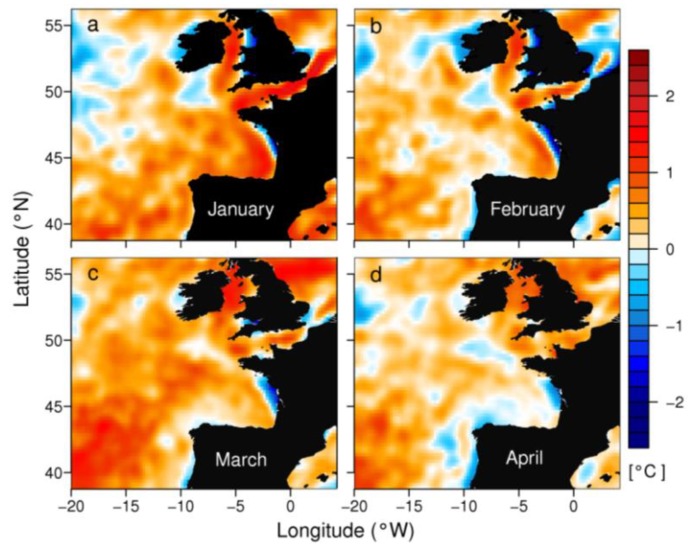
Monthly average Sea Surface Temperature (SST) anomalies observed in the North Atlantic region from January to April 2012.

At the mesoscale, positive SST anomalies were observed between the English Channel and western Iberia in winter (Figure 6), but negative anomalies appeared at a smaller scale near the coast due to positive Ekman transport in Galicia and deflected river plumes in the southeast Bay of Biscay.

### 2.2. Distribution of *D. acuminata* Populations in 2012

**Figure 7:**
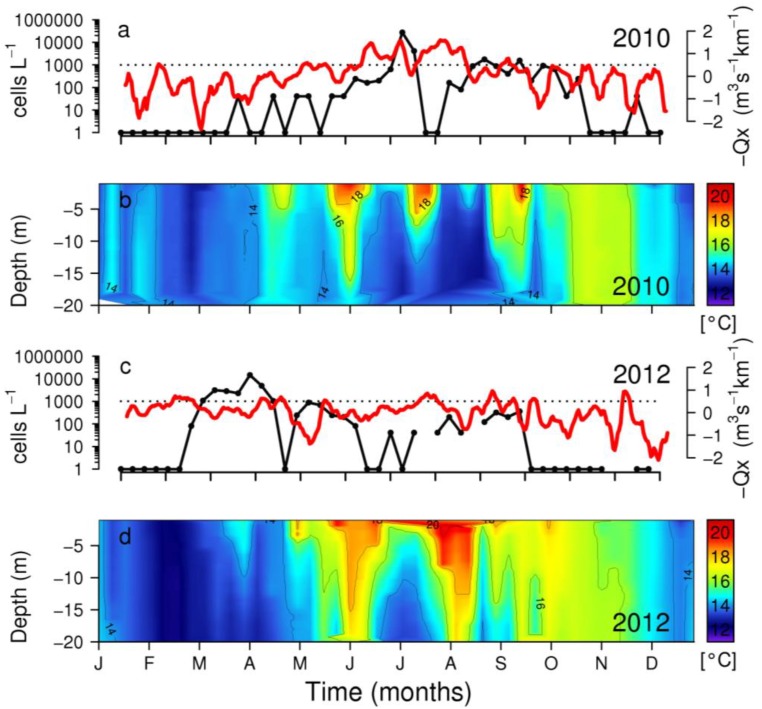
Weekly distribution of *D. acuminata* (black line), upwelling index (7-day running mean) (red line) and vertical distribution of temperature at station P2 in Ría de Pontevedra during (**a**,**b**) years of late (2010) and (**c**,**d**) early (2012) initiation of *D. acuminata* blooms.

**Figure 8:**
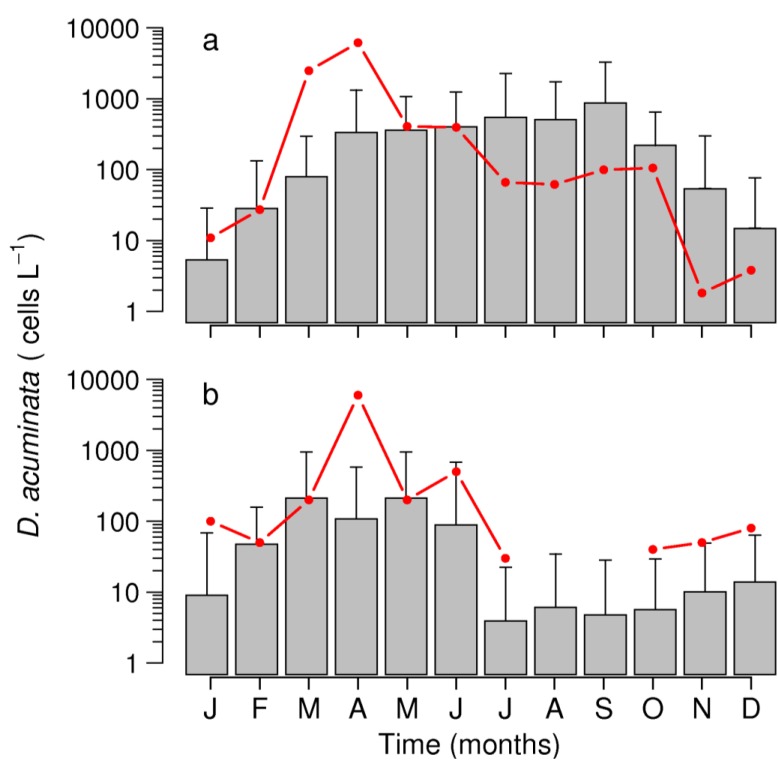
Monthly average *D. acuminata* densities during 2012 (red line) and mean (+SD) of the last 19-year (1993–2011 gray bar) in (**a**) Ría de Pontevedra (NW Spain) and (**b**) Arcachon Bay (southern Bay of Biscay).

During 2012, an extraordinary bloom of *D. acuminata*, in terms of early initiation and intensity, was observed in the Galician Rías Baixas. A maximum of 5.3 × 10^4^ cells L^−1^ in integrated (0–5 m) hose samples was found at the innermost station of Ría de Pontevedra during the last week of March (see Figure 7c and Section 2.3.1). Very intense blooms of *D. acuminata* were also observed the first week of April in the southern Bay of Biscay (SW France); a maximum monthly average of 3.6 × 10^4^ cells L^−1^ was found at the outermost station (Bouée) in Arcachon Bay (data not shown but included in the mean density estimates for the whole bay). These densities represent large positive anomalies compared with the 20-year means in both regions (Figure 8).

### 2.3. Interannual Variability of *D. acuminata*

#### 2.3.1. Ría de Pontevedra (NW Spain)

**Figure 9:**
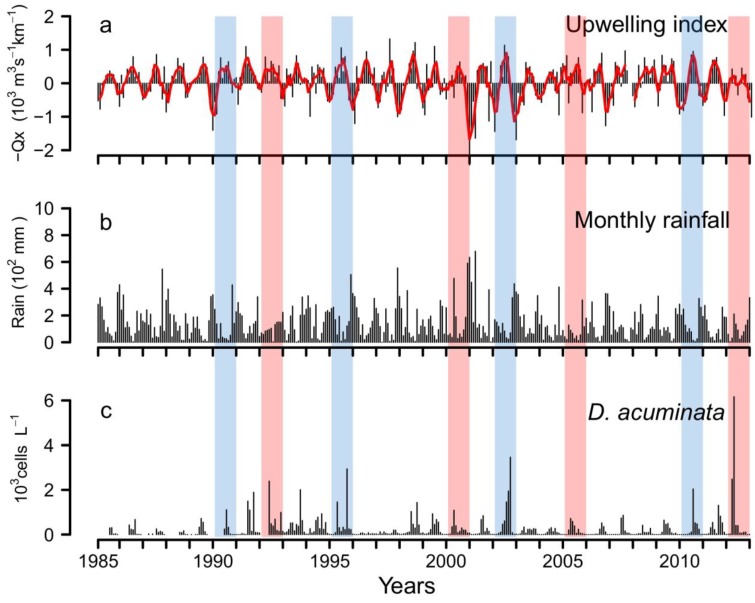
Interannual variability of (**a**) upwelling index, (**b**) monthly rainfall in Vigo and (**c**) monthly average of *D. acuminata* cells density in Ría de Pontevedra (NW Spain) from 1985 to 2012. Years with normal (blue) and anomalous (red) patterns are highlighted.

**Figure 10:**
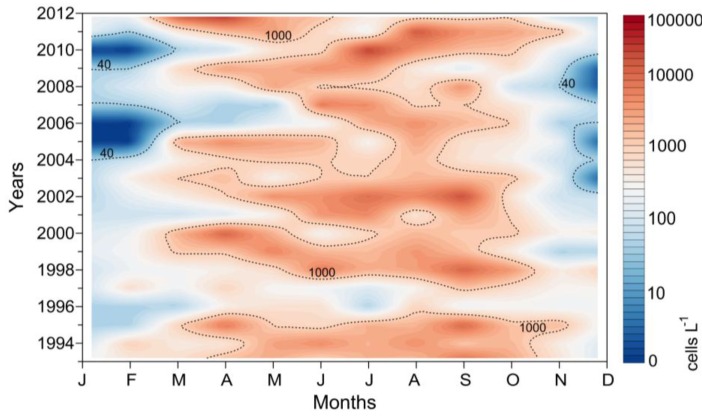
Average cell density of *D. acuminata* within the Ría de Pontevedra from 1993 to 2012. Dashed-line contours indicate 40 (detection level in the monitoring counts) and 1000 cells L^−1^ (bloom level). Note that 0 indicates no detection of overwintering cells even in the weekly vertical net-hauls

*D. acuminata* exhibited record densities (cell L^−1^) in 2010 and 2012 (7.4 SD), and minima in 1996 and 1997 over the 28-year time series (Figure 9). Analysis of the time-series provides evidence that years with early (March-April) bloom initiation—e.g., 2000, 2005 and 2012—coincided with anomalous patterns of Ekman transport the previous winter, *i.e.*, dominance of upwelling at a time of the year normally characterized by predominance of southerly winds and negative Ekman transport [22]. In contrast, a later (June–July) initiation of *D. acuminata* blooms—e.g., 2002 and 2010—coincided with years with normal upwelling patterns, *i.e.*, dominance of downwelling in the winter months (Figure 2, Figure 3). Another important observation is that, in addition to the anomalous winter upwelling conditions, years of early blooms of *D. acuminata* in Ría de Pontevedra showed persistence of scattered populations of this species inside the rías during the winter months (overwintering cells) (Figure 10). Further, the first peak of *Dinophysis* required the establishment of a slight thermal stratification following relaxation or downwelling after moderate upwelling pulses (Figure 7). 

#### 2.3.2. Arcachon Bay (SW France)

Highest average density found at one station in 2012 inside Arcachon Bay was 3.6 × 10^4^ cells L^−1^, which is 100 fold the average cell density of *D. acuminata* in the area at this time of the year. Maximal weekly densities of *D. acuminata* cells in Arcachon Bay (5 × 10^5^ cells L^−1^ in 2012) were always observed at station 7 located at the entrance of the Bay, a pattern that supports the view that these events originate in the open coastal waters. Samples taken 100 km south of Arcachon Bay showed already high *D. acuminata* densities on April 2 and 3, 2012 (from 5 × 10^2^ to 16 × 10^3^ cells L^−1^). Although the *D. acuminata* bloom in 2012 occurred at its regular time of the year (end March–April), cell densities were exceptionally high in Arcachon Bay and outside.

**Figure 11:**
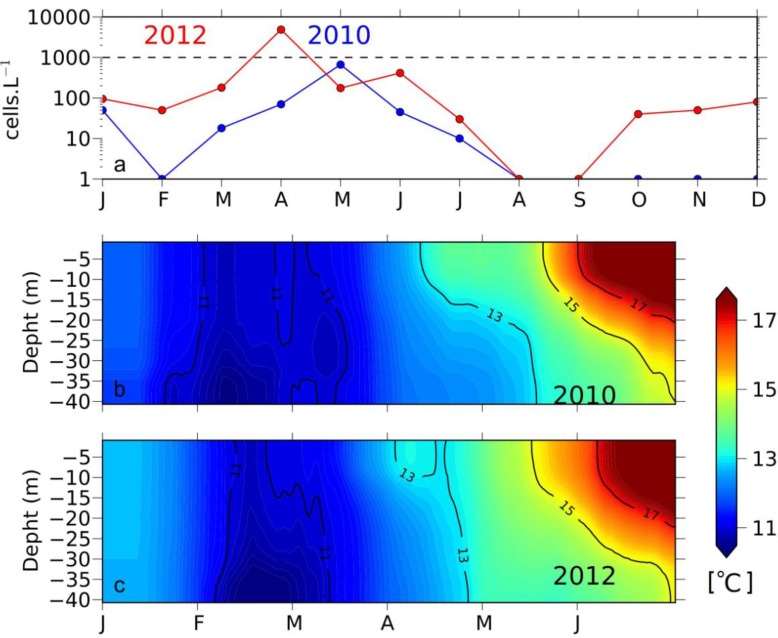
(**a**) Monthly average of *D. acuminata* distribution in SW France for 2010 and 2012; (**b**,**c**) temporal variation of temperature profiles inferred from MARS 3D simulations at the entrance of Arcachon Bay (1.44°W 44.67°N).

The dominance of anomalous northerly winds observed in February and March 2012 led to favourable upwelling conditions and thermohaline stratification due to the southward deflection of the Gironde plume. Comparison between a regular year, such as 2010 and 2012, showed that thermal stratification does not usually occur until late May, but was however established since early April in 2012 (Figure 11).

## 3. Discussion

### 3.1. Biological Conditions Preceding *Dinophysis* Blooms

Success in recent years to establish *Dinophysis* in culture led to understanding of its feeding behaviour. It is now well established that *D. acuminata* and other *Dinophysis* species are obligate mixotrophs which require live ciliate prey (*Mesodinium* species) and light for sustained growth [23,24,25]. Culture experiments have also shown the relationship between an ample supply of prey and high division rates in *Dinophysis* [24,26]. In field populations, *Dinophysis* may be subject to prey limitation most of the time and peak densities are preceded by or co-occur with high densities of *Mesodinium* [18,27]. Furthermore, *D. acuminata* and *Mesodinium rubrum* were observed exhibiting similar diurnal vertical migrations in a 24-h study under stratified conditions in Ría de Vigo [28], and thin layers of the dinoflagellate and the ciliate co-occurred in time, but not always in space, in a coastal inlet in the northern Baltic Sea [29].

In addition, the photosynthetic ciliate *Mesodinium* also depends on ingestion of live prey, cryptophytes of the genera *Teleaulax* and *Geminigera*, to sustain growth [30]. Koike *et al.* [31] monitored for three years the presence of cryptophytes with plastids that bound to oligonucleotide probes specifically designed against the *Dinophysis* plastidic ribosomal RNA. These authors found that in Okkirai Bay (NW Japan), high densities of probe-bound cryptophytes always occurred before seasonal peaks of *Dinophysis fortii*.

*Mesodinium* spp. are detected all year round off Ría de Vigo in low densities (Reguera, unpubl. data). Like *Dinophysis*, *Mesodinium* can survive without prey for months, but the population exponential growth will not take place under prey limitation [25]. Increased numbers of *Mesodinium* are not observed until May–June, following increased densities of *Teleaulax*-like cryptophytes. Red patches of the ciliate are frequent in July–August in the Galician Rías, but in 2012, red patches of *Mesodinium* were observed as early as March [32]. Unfortunately, quantification of *Mesodinium* spp. is not included in routine harmful algae monitoring programmes. Likewise, in the shelf waters off Arcachon Bay, *D. acuminata* growth will be controlled by the time-window of its prey growth and its availability to *Dinophysis* cells. The spring phytoplankton bloom in this region develops regularly from mid March to the beginning of May, with peak chlorophyll concentrations ranging from 2 to 4 µg L^−1^ [33]. This spring bloom is short-lived (less than one month) due to nutrient limitation in an area with no significant river inputs. In regular years, local upwelling processes and the spring bloom precede *D. acuminata* blooms in Arcachon Bay, which are restricted to the spring (April–May) and only in exceptional cases have been observed at other times of the year [34].

### 3.2. Anomalous Hydrodynamic Conditions Favouring Initiation/Intensification of *D. acuminata* Blooms in 2012

Increased stability and deepening of the photic zone along with initiation of thermal stratification off Arcachon Bay is synchronous with the spring (March) phytoplankton bloom. Local upwelling-promoting winds are south to south-westerly (Figure 4), and the advection of planktonic populations through upwelling, including *Dinophysis* populations, requires the previous establishment of stratification and a two-layered circulation pattern. Earlier studies have shown that even if thermal stratification is low at this time of the year, the Gironde plume can reach in a significant way the shelf break in the southern (<46°N) Bay of Biscay [35,36], including the shelf off Arcachon Bay. However, in 2012, anomalous winter patterns with dominance of northerly winds caused a deflection of the Gironde plume to the south and exceptionally long-lasting haline stratification. We can thus propose that during 2012, the unusual haline stratification in the southern Bay of Biscay between February and April permitted several upwelling processes leading to a sustained growth of *Dinophysis* off Arcachon Bay. Thus, although it is not unusual in this Bay to have a peak of *D. acuminata* in April, such exceptionally high densities of *Dinophysis* have never previously been observed in this area. We can speculate that these high cell numbers may have been the result of a successful recruitment of *Dinophysis*, further advected into Arcachon Bay, due to enhanced biological conditions (*i.e.*, prey abundance) on the adjacent shelf during the preceding winter in 2012.

In the Galician Rías, results here confirm the close coupling between initiation of the upwelling season and the onset of the *D. acuminata* growth season. Increased densities of cryptophyte populations and *Mesodinium* following upwelling-promoted nutrient enrichment and thermohaline stratification would establish the conditions for *Dinophysis* growth within the Rías. Upwelling pulses have been proposed in an earlier conceptual model [18] as a requisite to inoculate *Dinophysis* populations into the rías in years (such as 2007) when cells went undetected in the winter. Here, we show that the persistence of winter populations of *D. acuminata* within the rías is another factor that accompanies early blooms of this species in the Galician Rías. Therefore, the co-occurrence of persistence of overwintering population, anomalous predominance of upwelling in winter and the availability of suitable densities of ciliate prey appear as the suite of optimal conditions leading to high-density early blooms of *D. acuminata* in this area. 

### 3.3. Implications for Predictive Capabilities

Increased predictive capabilities of toxic microalgae outbreaks is a major objective of harmful algae experts from any region subject to their detrimental effects on aquaculture exploitations. In this context, one of the objectives of the ongoing European project ASIMUTH (“Applied Simulations and Integrated Modelling for the Understanding of Toxic and Harmful Algae Blooms”) is the identification of key past events which will be re-analysed and used for training the modelling system. One of the main gaps in our knowledge of the population dynamics of *D. acuminata* is to determine the causes of its interannual variability and in particular, the factors triggering early bloom initiation.

Results here provide important clues to the climatic anomalies leading to environmental conditions that promote the initiation of *D. acuminata* growth. Observations on the Galician time series support the conceptual model proposed by Velo-Suárez *et al.* [18]. In addition, the scattered winter populations observed in years when early blooms occurred is a third factor that combined with the anomalous winter upwelling patterns and suitable prey densities would lead to exceptional early spring blooms of *D. acuminata*.

Anomalous winter wind patterns with predominance of northerly winds must, therefore, be taken as a risk factor favouring early/intensified blooms of *D. acuminata* in both aquaculture sites of SW Europe, the Galician Rías and Arcachon Bay. Unfortunately, there are no regular weekly HAB monitoring programmes on the Spanish Basque and Cantabrian Sea coasts. Presumably, spring phytoplankton blooms in all northern Spain, and subsequently, the onset of *D. acuminata* populations, were influenced by the same mesoscale anomalies. High densities of *D. acuminata* in the ballast water of ships from the Spanish Basque coasts arriving to La Rochelle (located to the north of the Gironde estuary) in the spring of 2012 [37] support this view. 

Another risk factor preceding *Dinophysis* blooms that should be included in the observations of monitoring programmes is increased densities of *Teleaulax*-like cryptophytes and *Mesodinium.* Nevertheless, we have to bear in mind that the accurate identification of suitable cryptophyte preys for *Mesodinium* would require the use of molecular probes, and that there are several species of *Mesodinium* in field populations and only one of them (*M. rubrum*) has been tested as a suitable prey in *Dinophysis* cultures. Interestingly, early blooms of *D. acuminata* in the Galician Rías seem to “crash” early too and then, we may expect that anomalous winter-upwelling events will lead to early initiation and early decline of *D. acuminata* blooms and DSP outbreaks in Galicia and to intensification of spring DSP outbreaks in Arcachon Bay. The reasons for the early decline need to be explored but we can not discard an endogenous clock determining a maximum length of population growth per season.

Here, we present preliminary results that contribute to a better prediction of *D. acuminata* blooms in SW Europe, but further work is necessary. The response of *Dinophysis* populations to climate anomalies will not appear as a simple linear relationship but as an indicator of windows of opportunity as already described for pelagic fish populations in upwelling systems [38,39]. Our next steps will include: (i) Parameterization of the effect of these anomalies (intensity and persistence of winter wind anomalies) on the development of exceptional early spring blooms of *D. acuminata*; (ii) Analyses of time series to identify climate signals underlying the observed wind circulation anomalies*.*

## 4. Experimental Section

### 4.1. Meteorological Data

Data on wind speed and direction on the northwest and north Iberian slope were obtained from Seawatch buoys operated by the Deep Water Network (Silleiro, Vilano, Bares, Peñas and Bilbao buoys) of Puertos del Estado [40] and by the IEO-operated AGL buoy at Santander [41]. The average wind rose diagrams were constructed with data from the whole period since each buoy was moored: Bilbao in 1990, Bares in 1996, Silleiro, Vilano and Peñas in 1998 and AGL in 2007.

Time series of six-hourly Ekman transport (upwelling indexes) data over the last 28 years (1985–2012) were estimated using model data from the U.S. Navy’s Fleet Numerical Meteorology and Oceanography Centre (FNMOC) derived from Sea Level Pressure (www.indicedeafloramiento.ieo.es) on a grid of approximately 1° × 1° centred at 42.5°N 9.5°W, a data acquisition point representative for the Galician Rías. Monthly rainfall data from a meteorological station at Vigo airport (Peinador) (42°13.89′N, 8°37.29′W) were provided by the Spanish Meteorological Agency (www.aemet.es). Outflow of the Adour River and wind measurements (Pointe de Socoa, 43.4°N–1.69°W) were provided by Meteo France. Daily SST anomalies over SW Europe, between the English Channel and western Iberia, from January to April 2012 were taken from NOAA [42]. These data were used to estimate the monthly SST anomaly.

### 4.2. Phytoplankton Data

Weekly reports of phytoplankton distribution in Ría de Pontevedra from 1985 to 2012 were obtained from the IEO-Vigo HAB monitoring (1985–1991, data from three stations, June to December) and from the Galician Monitoring Program at INTECMAR (1992–2012, data from 10 stations, all year round). Plankton samples for quantitative analyses have been collected in the same way, since 1986 with a dividable hose sampler (recommended by the ICES Working Group on Exceptional Algal Blooms) [43] that samples the whole water column from 0 to 15–20 m (0–5, 5–10, 10–15, 15–20 m), and immediately fixed on board with acidic Lugol’s iodine solution [44]. In addition, vertical net-hauls (0–20 m) were performed and the samples transported alive to the laboratory for qualitative identification of species and detection of low-density harmful species that might go undetected with conventional counting methods. Phytoplankton data for Arcachon Bay (1988–2012) were obtained from the French HAB monitoring programme (REPHY, IFREMER) database [45]. Hydrobios bottle samples were collected at 5 m depth biweekly at 14 different stations between the south of the Gironde Estuary and the Spanish coast. 

Quantitative phytoplankton analyses in both countries were carried out according to the Utermöhl [46] method. Samples were counted after sedimentation (4–24 h) of 10–25 mL columns under an inverted light microscope fitted with 100×, 200× and 400× objectives and phase-contrast optics. To enumerate large species, such as *Dinophysis* spp., the whole surface of the chamber is scanned at a magnification of 100 × (detection level of 100–40 cell L^−1^).

Average cell densities of *D. acuminata* were used for the time series in the two study areas to smooth variability associated with tidal changes and local wind forcing.

### 4.3. Numerical

The MARS3D hydrodynamical model [47] was used to simulate the hydrodynamics in the southern Bay of Biscay. Model simulations extended from the French coast to 8°W and from the northern Spanish coast to Southern England. The horizontal mesh size used had a resolution of 4 km, and 30 vertical levels were considered with a finer resolution near the surface. The simulation was forced by real conditions of tide, river runoff, heat fluxes and wind using data from the Arpège model of the French Met Office. The model was spun up for one year prior to simulation of the observation period. It reproduced the main structures detected by remote sensing and *in situ* measurements of the Bay of Biscay during the study time-window.

## 5. Conclusions

Initiation of the growth season of *D. acuminata* in the Galician Rías is strongly linked to the upwelling season. Early initiation of the blooms, as was the case in 2012, is associated with anomalous predominance of upwelling the previous winter. In Arcachon Bay, model simulations showed increased haline stratification associated with southward advection of the Gironde plume that year. Anomalous wind patterns, with predominance of northerly winds during the preceding (2011–2012) winter appear as the common mesoscale meteorological driver that explains the exceptional blooms of *D. acuminata* and DSP outbreaks that hit aquaculture sites in Galicia and the southern Bay of Biscay in the early spring of 2012.
